# Translation and validation of the meat attachment questionnaire (MAQ) in a French general practice population

**DOI:** 10.1038/s41598-025-86270-x

**Published:** 2025-01-18

**Authors:** Bruno Delaunay, Benoit Tudrej, Augustin Bernard, Alexandra Dupuy, Claire Malavergne, Trystan Bacon, Paul Sebo, Hubert Maisonneuve

**Affiliations:** 1 University College of General Medicine, University Claude Bernard Lyon 1, Lyon, France; 2 University Institute for Primary Care (IuMFE), Faculty of Medicine, University of Geneva, Geneva, Switzerland

**Keywords:** Epidemiology, Psychology and behaviour

## Abstract

Meat consumption has significant implications for both individual health and the environment. Understanding individuals’ attachment to meat is crucial for designing effective interventions to reduce consumption. The MAQ is a tool developed to assess individuals’ attachment to meat. This study aims to translate and validate the MAQ into French for use in a general practice population in France. The study was conducted in three phases: translation, pretesting through cognitive interviews, and testing through a cross-sectional study of general practice patients. Descriptive, factorial, and internal consistency analyses were performed to validate the French version of the MAQ. The French version of the MAQ consists of 17 items in four dimensions: Hedonism, Affinity, Entitlement, and Dependence. Face validity was confirmed by cognitive interviews. The RMSEA and CFI were 0.06 and 0.92 respectively, showing acceptable goodness-of-fit. Internal consistency was demonstrated with Cronbach’s alpha and Loevinger’s H coefficients exceeding 0.7 and 0.3, respectively. The French version of the MAQ is a valid and reliable tool for assessing individuals’ attachment to meat in a general practice population. Its application shows promise for the design of targeted interventions to reduce meat consumption, benefiting both individual health and environmental sustainability.

**Supplementary Information:**

The online version contains supplementary material available at 10.1038/s41598-025-86270-x.

## Introduction

Meat products hold an important place in people’s diets worldwide. Average annual meat consumption per person worldwide is about 33 kilograms^1^, rising to 50–200 kg in high-income countries^1^ and 65 kg in France^2^. Between 1961 and 2022, the world’s population doubled, while global meat production quadrupled to 361 million tons^1^.

French and international recommendations suggest an annual consumption limit of 36 kg per person per year to maintain good health^3–5^. Meat is an important source of protein, iron and vitamins^6^. However, excessive consumption has been associated with increases in all-cause mortality^7,8^, cardiovascular mortality and cardiovascular events^9–11^, cancer (especially colorectal cancer)^12^, obesity and diabetes^11,13^. Given the difficulty in measuring the impact of excessive meat consumption on human health, these consequences may be underestimated^14,15^.

Excessive consumption also leads to overproduction, which has significant environmental impacts in terms of greenhouse gas emissions, soil and water acidification, eutrophication of aquatic environments (asphyxiation of the aquatic environment by excessive inputs of nutrients such as nitrogen and phosphorus)^16^, water consumption^17,18^, use of fertilizers, loss of biodiversity, air pollution^18^, deforestation^19^ and climate change^17–19^. An individual’s carbon footprint (i.e., the amount of greenhouse gases produced by one person) could be reduced by a factor of 2 by adopting a vegetarian and local diet. This individual action could have the greatest impact on reducing CO2 emissions^20^.

In terms of planetary health, reducing meat consumption benefits both human health and the environment. This is known as a cobenefit^21^.

Several authors have developed models and theories to support the construction of interventions aimed at influencing the behaviour of meat consumers. For instance, Graça, et al.^22^, developed the construct of meat attachment, which is conceptualized as a relatively stable individual characteristic rather than a transient state. Meat attachment comprises four principal dimensions: (i) ‘hedonism’ i.e. meat as a source of pleasure, (ii) ‘affinity’ as opposed to feelings of repulsion towards meat consumption, (iii) feelings of ‘dependence’ on meat consumption, and (iv) ‘entitlement’, which measures feelings of entitlement towards meat consumption. The Meat Attachment Questionnaire (MAQ) was developed to measure this construct. It consists of a reflexive psychometric scale including 16 items which were answered using a 5-level Likert scale, exploring the 4 dimensions previously mentioned dimensions^22,23^. The English and Portuguese versions of the MAQ have been validated in the general population^22^.

Meat attachment acts as a moderator of how individuals respond to interventions aimed at reducing meat consumption. For example, individuals with a MAQ score reflecting a high meat attachment are less likely to engage with messages promoting a plant-based diet, as their attachment moderates their willingness to change their dietary habits^22,23^.

The MAQ has been used in various studies in the field of meat consumption and related behaviours. These studies assessed either (i) the impact of graphic awareness messages^24^, (ii) the visual attractiveness of vegetarian substitutes^25^, (iii) consumers’ demand for vegetarian steaks (Bryant et al., 26), (iv) the evolution of representations about meat^26^, and (v) the intensity of ‘Meat Paradox’ i.e. wanting to eat meat but being opposed to animal suffering^27^. The MAQ has been shown to predict intentions and willingness (openness to the possibility of engaging in the behaviour) to reduce meat consumption^22,23^.

This makes the MAQ a valuable tool for developing tailored interventions in general practice aiming at meat consumption reduction depending on different attachment profiles, as general practitioners (GPs) have the potential to be major contributor to the reduction of meat consumption at the individual level. Moreover, its brief completion time (less than five minutes) makes it suitable for use in time-constrained settings. Prior to implementing it in general practice in French-speaking countries, it was necessary to translate the MAQ into French and to verify the validity of this translated version in a general practice population.

## Method

### Study design

**Fig. 1:**
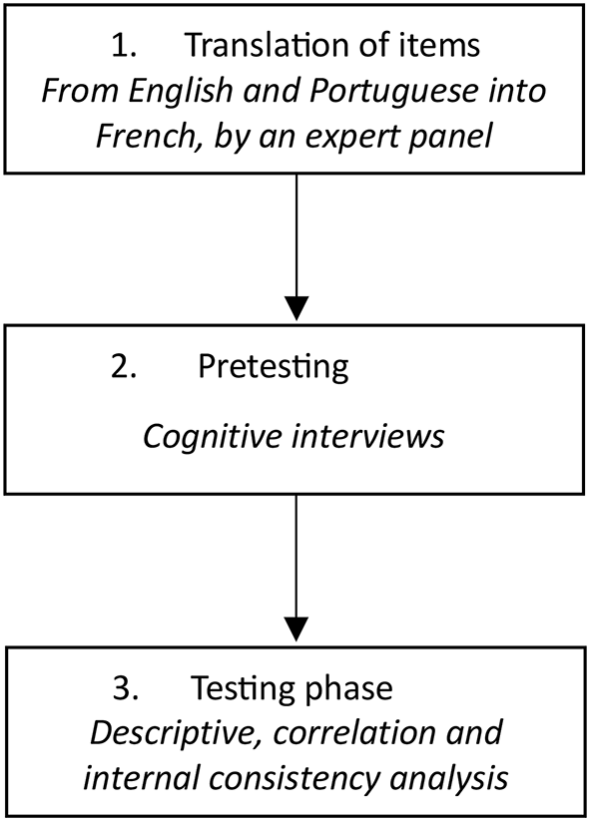
Translation and validation phases of the MAQ questionnaire into French.

The aim of our study was to translate in French and validate a questionnaire: the Meat Attachment Questionnaire (MAQ). We carried out the study in three phases (Fig. 1).

In the first phase, we translated the MAQ^28^.

In the second phase, we verified the face validity of the MAQ through a qualitative study using cognitive interviews^29^ with general practice patients.

In the third phase, we confirmed the validity of the French version of the MAQ by conducting a cross-sectional study of general practice patients.

### Study context

We conducted our study in France, in the Rhône-Alpes region, in 2023 among a population of adult general practice patients.

This study is part of a Franco-Swiss planetary health project aimed at developing different tailored interventions to reduce meat consumption among general practice patients. To achieve this, we first needed a reliable tool to identify the level of attachment to meat (score), the profiles associated with the level of attachment and the dimensions that have the greatest influence on the overall score. In a second step (not presented in this article), we planned to use CIBER analyses to correlate scores and profiles with varying levels of action and intentions to reduce meat consumption.

#### Translation

We supervised a double translation from Portuguese and English into French, as the MAQ was designed in Portuguese and then translated into English by the same authors. This translation took place in several phases^28^ to obtain a literal translation and then a transcultural validation of the MAQ^30^.

The first step was translation from English into French by a professional translator (VB) and a native English speaker (AT) and from Portuguese into French by a native Portuguese speaker (BT).

The translations were then reviewed by a committee of four English- and Portuguese-speaking researchers (DHH, JHR, JS, BT). As far as possible, we took everyone’s comments into account and discussed them within the team to reach a consensus when opinions differed^31^.

We then performed back-translations into English and Portuguese by a professional translator (CH), a native English speaker (CMa) and a native Portuguese speaker (SB) who were not involved in the first phase^28^.

We maintained contact with the authors of the original version of the MAQ throughout the process to clarify any possible concerns. We submitted our translations and back-translations to the extended research team and to the authors of the original MAQ to ensure their concordance (JG)^28^.

#### Pretesting: cognitive interviews

Cognitive interviews were used to explore face validity. The aim of this phase was to confirm that respondents’ understanding of item meanings was similar to the intended meaning^31^. This phase was also an essential part of the process of cross-cultural translation validation^30^.

The methodology of this phase complied with the COREQ quality criteria (Consolidated Criteria for Reporting Qualitative Research)^32^.

##### Population

The target population were patients consulting a general practitioner (GP) with a large range of characteristics including age, sex, socioprofessional category, and quantity of meat consumed per week, to ensure maximum diversity in responses.

To obtain a sample reflecting the target population, we used a snowball sampling technique based on a convenience sample^33^.

Initially, we selected participants from our own circle (who were also patients of a GP) and inquired them if they knew other participants we could contact. We then contacted them by phone, email or social media.

The literature does not clearly establish the required number of interviews needed to reach data saturation, but it is generally agreed that conducting 5 to 15 interviews is sufficient. We considered data saturation to have been reached when no further data emerged after two or three interviews^34^.

The participants provided informed consent before the interview, and they were informed of their right to withdraw at any point during the interview.

##### Data collection and coding

AD, BD, and CM conducted the cognitive interviews^35^. It was agreed that a single face-to-face interview would be carried out with each participant in the absence of a third party.

Each question required the participant to read the item aloud and verbalize their thoughts and the reasons for their response using the think-aloud technique^29^.

Next, we asked more specific questions, known as probes^34^, to explore particular dimensions of understanding. These questions were selected from the literature^29^ and adapted as the interviews proceeded, for example, to explore redundancy (e.g., ‘Do you find that this question is almost similar to another question I asked you? ‘), to clarify the meaning of a word or phrase (e.g., ‘How do you understand the meaning of.? ‘), or to assess whether a question was offensive (e.g., ‘Do you find this question offensive? ‘).

Before conducting the interviews, we recorded all the above elements in a self-developed interview guide. It also included sociodemographic characteristics to be collected and examples of sentences to be used at the beginning or end of the interview (primers, closing sentences). This approach enabled us to structure the interviews effectively and ensure their reproducibility within the research group (See Supplementary material 1).

We recorded the interviews with a voice recorder in combination with handwritten notes and then transcribed and coded the verbatims using a 6-point coding grid (See Supplementary material 2). We coded these responses as appropriate (code 1) or inappropriate (codes 2 to 6). For example, 2 = ambiguous response; 6 = nuanced response). Code 5 (interesting answer) was the only one that overlapped with the other codes, so that an item could be coded twice.

We considered an item to be satisfactory if its adapted response rate (code 1) was greater than 85%^29^.

##  Testing phase: confirmatory factorial analysis and internal consistency analysis

The methodology for this phase was done in accordance with the Strengthening the Reporting of Observational Studies in Epidemiology (STROBE) quality criteria^36^.

### Population

The target population was represented by adult patients of any genders consulting a GP (inclusion criteria).

The exclusion criteria included patients younger than 18 years, patients who did not understand French, and/or patients who were not able to give consent.

The practices were selected using a cluster random sampling technique^37^, stratified by the place of practice among the Rhône-Alpes region. For this purpose, we used the freely available 2020 health directory lists of all healthcare professionals^38^, from which we selected the general practices of the Rhône-Alpes region.

We aimed to recruit 800 patients (see the “Statistics” section) and planned to administer 20 questionnaires per practice. We therefore set the targeted number of practices to 40 and, anticipating 10% participation, randomized 400 practices to account for possible refusals or failures. We stratified our sample using quotas based on the proportion of practices in each department (i.e., Ain, Ardèche, Drôme, Haute-Savoie, Isère, Loire, Rhône, Savoie)^38^.

### Contact methods

From January to March 2023, we contacted first by phone^39^ and second by email each randomly selected practice until we met the required number of GPs per department (see the “distribution and data collection” section).

If they agreed to participate, we contacted them again to arrange a date for their visit following the randomization protocol explained below. If they refused or if we received no response after three consecutive reminders, we contacted the next practice on the list. We excluded practices that could not be contacted (change of address, end of practice, unassigned number) and practices whose main activity was not general practice.

### Data collection

We randomized the practices and the day of the visit by a third party and visited (AD, CM, BD, AB) the waiting rooms of the recruited practitioners.

We distributed the questionnaires to all participants who met the inclusion criteria and met no exclusion criteria. We then asked patients who provided their consent to complete the French version of the MAQ and who stayed in the waiting room to provide further explanations if needed.

### MAQ questionnaire

The French version of the MAQ designed for distribution to patients comprises 4 dimensions (Hedonism, Affinity, Entitlement and Dependence) divided into 17 items (one more item than the original questionnaire, see below for further explanations), randomly distributed within these dimensions to limit data collection bias (see supplementary material 3).

Responses to the 17 questions were given on a five-point Likert scale ranging from 1, strongly disagree, to 5, strongly agree.

Sociodemographic data, including year of birth, gender, postal code of residence and socio-professional category, were collected in accordance with the methodology employed by the French National Institute of Statistics and Economic Studies (INSEE).

### Statistics

#### Number of participants

Since descriptive analyses require a smaller number of participants^28^, we calculated the number of participants to include on the basis of the power required for factorial analyses. Recommendations for factorial analyses do not clearly state the methods for calculating the number of participants to be included^40^. However, it is generally accepted that between 300 and 500 participants are sufficient to accurately determine the correlation factors among variables^28,41^. Considering the sampling method described above, adjustment for clustering, and potential difficulties in contacting doctors, we decided to recruit 20 participants per practice from 40 practices, for a total of 800 participants.

#### Descriptive analysis

For each item, we calculated the mean and standard deviation.

The MAQ score is calculated by measuring the average response to the items, by dimension and in total^22^ (See Supplementary material 4). The score for each dimension and the total score therefore vary from 1 to 5. Each item has the same weight, except for items 15 and 16 (resulting from the division of item 15 into 2 items, following the cognitive interviews), whose score was divided by 2.

This choice maintained the average of 16 weighting points presented in the Portuguese and English versions of the MAQ, thus maintaining its comparability. The items coded inversely for measuring the subscores and the total score are items #4, #6, #9, #13, and #14 (5 equals 1, 4 equals 2, 3 remains unchanged, 2 equals 4, and 1 equals 5).

We considered that an item with 95% similar responses was not discriminative and should be deleted^42^.

#### Correlation analysis

We used the validscale command^43^ in Stata (StataCorp. 2020. Stata Statistical Software: Release 17. College Station, TX: StataCorp LLC https://www.stata.com) to assess the psychometric properties of the MAQ using classical test theory (CTT). The CTT is a widely used framework for assessing the psychometric properties of measurement instruments and is particularly suitable for validating questionnaires^44^.

To select the most relevant factors and organize them into coherent dimensions, we conducted a reliability study using confirmatory factor analysis (CFA) combined with goodness-of-fit indices. To assess the relevance of the statistical model, we used the root mean square error of approximation (RMSEA) and the comparative fit index (CFI).

These indices assess the fit between observed and expected data according to the specified model. An RMSEA < 0.06 and a CFI > 0.90 are generally considered to indicate a good fit^45^.

We used the convdiv option to assess convergent and divergent validities by examining a correlation matrix^43^.

#### Internal consistency

Finally, the internal consistency of the MAQ was validated by calculating Cronbach’s alpha and Loevinger’s H^46^. These are the most commonly used tools for measuring internal consistency in psychometric studies. We used these coefficients to verify the internal consistency of each item within the four dimensions^47^.

A minimum value of 0.70 for Cronbach’s alpha and 0.30 for Loevinger’s H coefficient was considered acceptable^48,49^.

#### Ethics

We obtained the agreement of an ethics committee on 03/01/2023 and filed an MR004 declaration with the CNIL. All methods were performed in accordance with the declaration of Helsinki and relevant guidelines and regulations, in particular, informed consent was obtained from all participants.

## Results

### Translation

The translation of the MAQ questionnaire into French resulted in a 16-items version with 4 dimensions. No significant issues were encountered during the process, and the original meaning of each item was preserved. The back-translation was submitted back to its original authors, who confirmed its accuracy. Supplementary material 5 details the first version of the MAQ translated into French.

### Pretest

AD, BD and CM contacted 11 individuals, all of whom agreed to participate. Each were given one interview, during an average of 30 min each. Each individual was interviewed face to face, in an environment of their choosing : home, workplace, neutral location or by video-conference. No third party was present at the time of the interviews. All participants were French, mostly from urban area, with an average age of 40 years old, 64% of them being women. The sociodemographic characteristics of the participants are detailed in Table 1.

**Table 1:** Socio-demographic characteristics of participants interviewed during the cognitive interviews. * : participant’s home.

Interview	Location	Sex – Age	Socio-professional category	Place of residence	Meat consumption (frequency)	Duration (minutes)
B1	Video call	M – 65	Engineer	Pouancé (49)	2/week	42
B2	Hospital	F – 46	Caregiver	Lyon (69)	8/week	41
B3	Video call	M – 29	Architect assistant	Brest (29)	1/week	33
A1	Outside	F – 28	Medicine resident	Lyon (69)	3/week	32
A2	Home*	M – 28	Medicine resident	Paris (75)	5/week	16
A3	Hospital	F – 55	Nurse	La Verpillère (38)	1/week	19
A4	Home*	F – 55	Physiotherapist	Colmar (68)	5/week	19
A5	Hospital	F – 21	Medical student	Lyon (69)	1/week	19
C1	Home*	F − 28	Medicine resident	Valvignères (07)	1/week	29
C2	Video call	F − 60	CNRS researcher	Paris suburbs (91)	3/week	59
C3	Home*	M − 26	Craftsman	Valvignères (07)	5/week	25

Of the 16 MAQ items, 14 were kept.

The other two items received only two “appropriate responses” (Table 2). We therefore reworked their composition and wording. These adjustments were made in accordance with the participants’ comments during the interviews.

**Table 2:** Frequency of code appearance per item in the first 7 interviews (V1 : before modification).

Item	Appropriate response	Ambiguous response	Redundant response	Offensive response	Informative response	Qualified response
Item 1	7					
Item 2	7					
Item 3	6				1	1
Item 4	7					
Item 5 (V1)	2		5			
Item 6	5			2		
Item 7	6			1		1
Item 8	6		1			
Item 9	7					
Item 10	2		5			
Item 11	7					
Item 12	7					
Item 13	6	1				
Item 14	7					
Item 15 (V1)	2	4			1	
Item 16 (V1)	7					

Supplementary material 6 details the verbatims of interest.

For example, item 5 (“I love eating meat”; “J’adore manger de la viande”) was deemed to be similar to items 1 and 10 (verbatims B1.1, B2.1, A1.1, and C1.1) by participants. Item 15 was therefore amended to:

“I love meals with meat” (“J’adore les repas avec de la viande”).

Item 15 (“eating meat is a natural and indisputable practice”; “Manger de la viande est une pratique naturelle et indiscutable”) included two different notions: natural and indisputable (verbatims B1.2, B2.2, A3, C1.2, C2). We therefore split the items in two, leading the total number of items to 17, in agreement with JG, the initial authors.

It should be noted that items 3 and 7 sometimes led to nuanced responses due to questioning about the notion of “right” (verbatim B1.3 and B1.4).

Item 6 was considered offensive on two occasions (verbatims A1.3 and A2). However, this did not result in any changes due to the sufficient number of appropriate responses.

Finally, item 10 was considered redundant with items 5 and 15, but only before they were modified (verbatims A1.1, C1.1). We therefore did not need to reword it. Of the seventeen items of the modified MAQ proposed in the last four interviews, all received three to four appropriate responses (Table 3). We therefore considered them to be clear to the population concerned. Supplementary material 3 details the modified French version of the 17-item MAQ.

**Table 3:** Frequency of code appearance per item in the last 4 interviews (V2 : after modification).

Item	Appropriate response	Ambiguous response	Redundant response	Offensive response	Informative response	Qualified response
Item 1	4					
Item 2	4					
Item 3	4					1
Item 4	4					
Item 5 (V2)	4					
Item 6	4					
Item 7	3		1			
Item 8	4					
Item 9	4					
Item 10	4					
Item 11	4					
Item 12	4					
Item 13	4					
Item 14	4					
Item 15 (V2)	4					
Item 16 (V2)	4					
Item 17 (= 16 from V1)	4					

### Testing phase

**Fig. 2:**
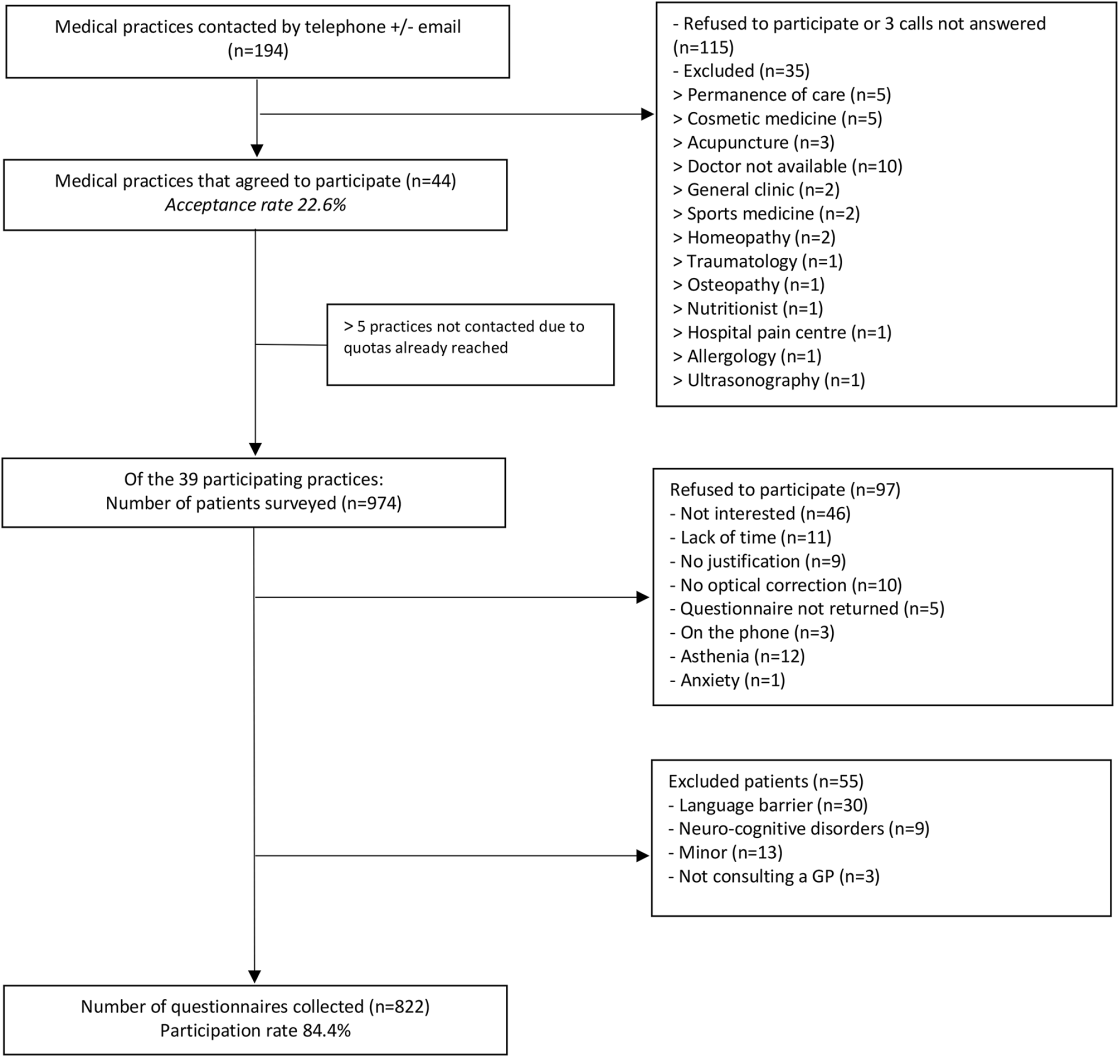
Patient inclusion flow-chart.

Figure 2 illustrates the study’s patient inclusion flowchart. Out of 194 eligible practices, 44 (22.6% participation rate) agreed to participate, while 115 practices declined or did not respond after three calls, and 35 were excluded.

Ultimately, we only visited 39 of the 44 practices originally planned, as the number of subjects to be included had already been reached. We interviewed 974 patients but 97 declined, and 55 were excluded. A total of 822 patients took the questionnaire (84.4% participation rate). A total of 822 questionnaires were analysed (816 without missing values, and 6 with 1 or more missing values).

Table 4 details the characteristics of the participants, who were primarily urban (65%), had a median age of 52 years (IQR = 31, min–max = 20–93), and were predominantly women (65%). The main socioprofessional categories were retirees (32.1%), employees (27.6%), and managers (20.2%).

**Table 4:** Socio-demographic characteristics of participants (*N* = 822).

Characteristics [*N* = 822]	Sample	Median (IQR, min-max)	Proportion of respondents [%]
Sex [*n* = 821]			
Female	536		65.29
Male	285		34.71
Non-binary	0		0
Age [*n *= 819]		52 (31, 20–93)	
< 40	239		29.19
40–60	267		32.60
>= 60	313		38.22
Place of residence :			
Urban area	534		65
Rural area	288		35
Socio-professional category (INSEE) (*N* = 822)			
Farmer	3		0.36
Craftsmen and women; business owners > 10 people	25		3.04
Managers and higher intellectual professions	166		20.19
Intermediate occupations	41		4.99
Employees	227		27.62
Manual workers	28		3.41
Retired	264		32.12
No activity	68		8.27

The descriptive statistics in Table 5 show that the average item scores were 2.4 to 4.2, with no floor or ceiling effects. For item 13,53% of respondents “completely agree[d]”. 6 questionnaires out of 822 were incomplete, explaining the different “n” values.

**Table 5:** Descriptive characteristics of each item submitted to 822 general practice patients during the test phase.

*N*°	Item	*N*	Mean (SD) [IC 95%]	Mean with inverse coding
1	Manger de la viande est un des bons plaisirs de la vie	822	3.74 (1.09) [3.67–3.82]	
2	Rien ne peut remplacer la viande dans mon alimentation	821	2.45 (1.22) [2.36–2.53]	
3	Du fait de notre place dans la chaîne alimentaire, nous avons le droit de manger de la viande	820	3.49 (1.14) [3.41–3.57]	
4	Je me sens mal à l’idée de manger de la viande	819	1.98 (1.16) [1.90–2.06]	4.02
5	J’adore les repas avec de la viande	822	3.48 (1.13) [3.40–3.56]	
6	Manger de la viande est irrespectueux de la vie et de l’environnement	820	2.40 (1.17) [2.32–2.48]	3.60
7	Manger de la viande est un droit incontestable de chaque personne	819	3.50 (1.24) [3.41–3.58]	
8	Rien ne vaut un bon steak	820	2.99 (1.26) [2.91–3.08]	
9	Une alimentation sans viande me conviendrait très bien	820	2.96 (1.26) [2.88–3.05]	3.04
10	Je raffole de la viande	819	2.93 (1.22) [2.85–3.01]	
11	Si je ne pouvais pas manger de viande, je me sentirais faible	819	2.36 (1.15) [2.28–2.44]	
12	Si on m’obligeait à cesser de manger de la viande, je serais triste	820	2.78 (1.37) [2.69–2.88]	
13	La viande me fait penser à des maladies	819	1.80 (1.03) [1.73–1.87]	4.20
14	En mangeant de la viande, je pense à la mort et à la souffrance des animaux	819	2.31 (1.23) [2.22–2.39]	3.69
15	Manger de la viande est une pratique naturelle	819	3.67 (1.03) [3.60–3.74]	
16	Manger de la viande est une pratique indiscutable	816	2.76 (1.14) [2.69–2.84]	
17	Je ne me vois pas ne pas manger de viande régulièrement	817	2.93 (1.28) [2.85–3.02]	

**Fig. 3:**
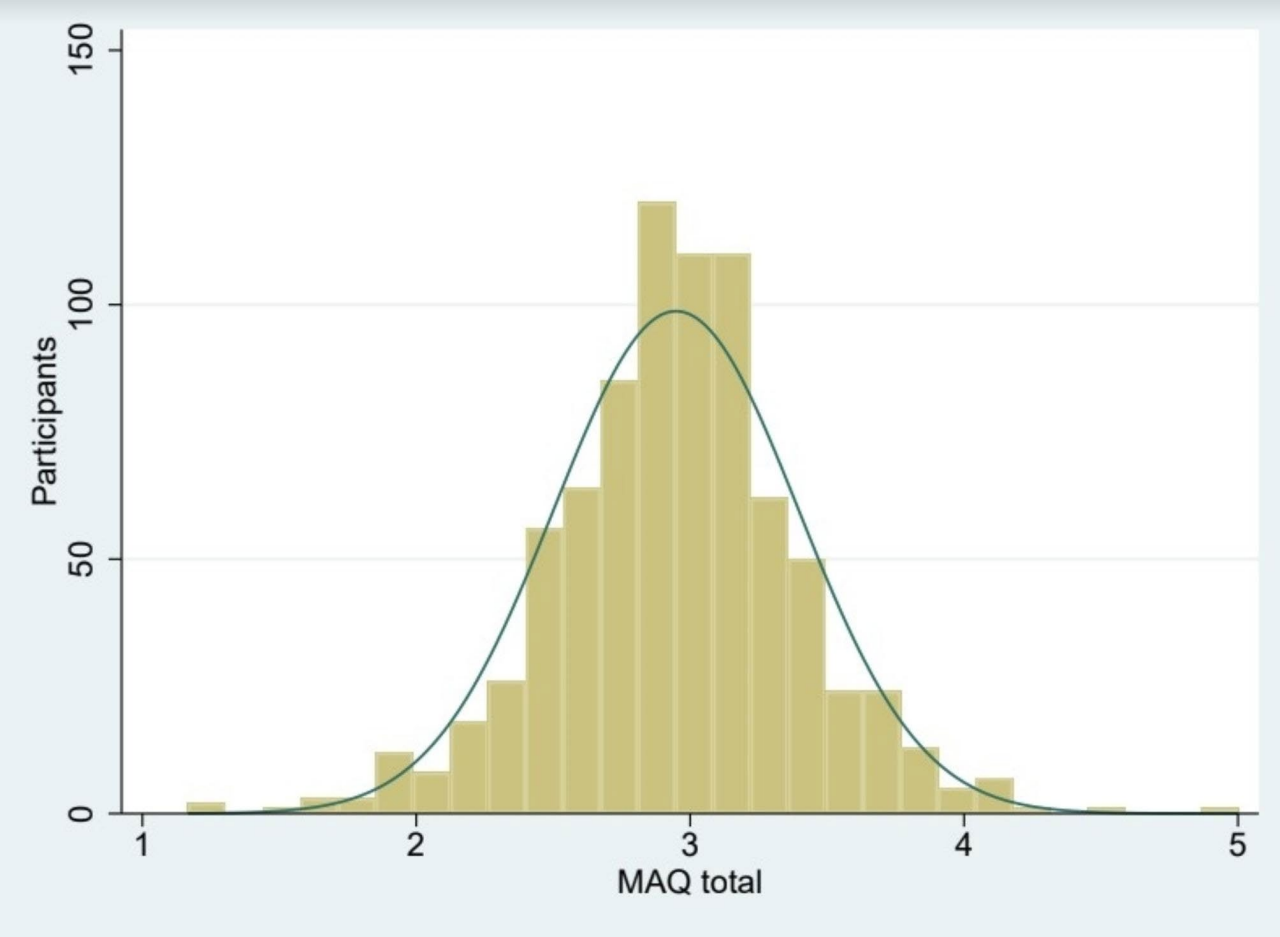
MAQ total score distribution chart.

We obtained a mean MAQ score of 2.95 (SD = 0.45), with a normal distribution, for a score ranging theoretically from 1 (low attachment to meat) to 5 (high attachment to meat) (Fig. 3).

Table 6 details the confirmatory factor analysis results, with the correlation coefficients per item and per factor. These factors helped us identify 4 factors. The RMSEA and CFI were 0.06 and 0.92, respectively, indicating an acceptable fit.

Each of the 4 factors included items whose themes were consistent. Factor 1 (“Hedonism”) combined items 1, 5, 8 and 10. Factor 2 (“Affinity”) combined items 4, 6, 13 and 14. Factor 3 (“Entitlement”) included items 3, 7, 15 and 16. Finally, Factor 4 (“Dependence”) combined items 2, 9, and 11 and 12 and 17. The French translations are available in Supplementary material 4. Notably, most items, excluding 4, 6, 7, 13, and 14, demonstrated correlations with multiple factors. We allocated them to the dimension with the strongest correlation or the most conceptually consistent meaning, a categorization consistent with the MAQ original study of Graça et al.^22^.

**Table 6:** Confirmatory factor analysis for the French version of the four-dimensional MAQ-17 questionnaire (*n* = 822).

Items	Factors*			
	1	2	3	4
1 Manger de la viande est un des bons plaisirs de la vie	0.797			0.499
5 J’adore les repas avec de la viande	0.839		0.436	0.527
8 Rien ne vaut un bon steak	0.803		0.497	0.590
10 Je raffole de la viande	0.854		0.439	0.602
4 Je me sens mal à l’idée de manger de la viande	0.414	0.746		
6 Manger de la viande est irrespectueux de la vie et de l’environnement		0.714		
13 La viande me fait penser à des maladies		0.692		
14 En mangeant de la viande, je pense à la mort et à la souffrance des animaux		0.778		
3 Du fait de notre place dans la chaîne alimentaire, nous avons le droit de manger de la viande	0.411		0.728	
7 Manger de la viande est un droit incontestable de chaque personne			0.737	
15 Manger de la viande est une pratique naturelle	0.400		0.727	
16 Manger de la viande est une pratique indiscutable	0.400		0.736	0.447
2 Rien ne peut remplacer la viande dans mon alimentation	0.532			0.717
9 Une alimentation sans viande me conviendrait très bien	0.509	0.465		0.707
11 Si je ne pouvais pas manger de viande, je me sentirais faible	0.430			0.721
12 Si on m’obligeait à cesser de manger de la viande, je serais triste	0.553		0.405	0.785
17 Je ne me vois pas ne pas manger de viande régulièrement				0,617
* factors obtaining more than 0.4 are displayed				

Table 7 demonstrates the internal consistency of the four-dimensional model, with Cronbach’s alpha and Loevinger’s H coefficients exceeding 0.7 and 0.3, respectively (0.84 and 0.61 for Hedonism, 0.72 and 0.42 for Affinity, 0.71 and 0.41 for Entitlement, and 0.75 and 0.40 for Dependence).

**Table 7:** Internal consistency (Cronbach’s alpha and H. Loevinger’s coefficients) by MAQ dimension.

Factors	Dimension	*N*° Item	Cronbach’s Alpha (*N* = 822)	H. Loevinger
F1	Hedonism*	1 ; 5 ; 8 ; 10	0.84	0.61
F2	Affinity*	4 ; 6 ; 13 ; 14	0.71	0.42
F3	Entitlement*	3 ; 7 ; 15 ; 16	0.71	0.41
F4	Dependence*	2 ; 9 ; 11 ; 12 ; 17	0.75	0.40

## Discussion

### Main results

We translated and verified the face validity and the psychometric validity of the French version of the MAQ in a sample of general practice patients in the Rhône-Alpes region.

The modified French version contained 17 items, one more than the original version, divided into four dimensions (Hedonism, Affinity, Entitlement and Dependence).

### Comparison with the literature

Our French version of the MAQ confirms the validity and internal consistency of the results obtained by the authors of the original version of the MAQ^22^. The 4 dimensions observed were similar to those from the original version (Hedonism, Affinity, Entitlement and Dependence)^22,23^.

The 2 items that we modified (item 15 and 5 of the original questionnaire) did not seem to cause any comprehension problems in the validation studies^22,23^, but the authors did not perform a face validation for these items. Furthermore, in our study, the coefficients assessing correlations and internal consistency were generally weaker and less precise. These differences may be explained by the selection of the study populations. Participants in Graça et al.‘s study^22^ were likely more informed about meat consumption, as they self-selected based on the survey title. Linguistic variations during translation might also contribute to these differences.

The total MAQ score in our study was lower than that reported by Graça et al. As women tend to have lower MAQ scores^22,23,50,51^, we hypothesize that this difference may be linked to the greater proportion of women in our study.

Our translation methodology, following the guidelines of Guillemin and Sousa^28,30^, involved literal translation followed by transcultural adaptation. The Delphi method^52^ was deemed unnecessary because it focuses on expert agreement, which was not essential for our questionnaire aimed at general practice patients.

We tested face validity through cognitive semistructured interviews^29^ using the “think aloud” and “probing” techniques^34^. It is also possible to use either directive or free interviews. In our study, the first was not suitable, as we needed to explore the response mechanisms to the items. The second tends to be used when the respondents are experts on the subject and was not suitable for our sample or for the target population of our questionnaire, as mentioned above.

The MAQ has been used in numerous studies^22,23,51^, alone or in combination with other questionnaires in the general population. However, our study is the first to translate and validate it in a general practice population in the French context.

### Strengths and weaknesses

The main strength of our study is its robust methodology. We followed the COREQ criteria^32^ for the qualitative phase and the STROBE^36^ for the quantitative phase. Our translation process followed validated protocols^28,30^ through two stages of literal translation and transcultural validation, with the help of a committee of experts and professional translators. MAQ authors were included in the process and validated the proposed modifications.

We performed face validation on a diversified sample, enabling us to test the comprehension of the items across various patient profiles. We developed an interview guide and a coding grid based on the literature^29,31^. The number of cognitive interviews was adequate^34^, which enabled us to recruit participants until data saturation.

We performed the third phase of psychometric validation on a large sample of patients with various sociodemographic characteristics, meeting the required number of participants^28,40^. Using a reliable database^38^, we randomized the practices and days of the week by a third party to avoid selection bias. We performed on-site visits, improving the quality of MAQ completion and resulting in high patient participation rates and a low proportion of missing data. We performed descriptive, factorial and internal consistency analyses similar to those of the validation source studies^22,23^.

Another strength of our study is the quality of the MAQ. Its English and Portuguese versions have interesting intrinsic qualities that we were able to preserve in our French version, thanks to our prior translation and face validation phases. The MAQ is concise, with clear items and a precise likert scale, making it easy to complete and analyse, as our results show. Its feasibility could be further improved with a shorter version, potentially using a multi-stage strategy.

#### Our study also has limitations

First, our sample included more women (65%), more managers and higher intellectual professions (34%) and fewer intermediate professions (8.4%) than did other general practice studies (58%, 13.5% and 16%, respectively, in a previous study^53^). These differences appear to have had a limited influence on the validation of the French version of the MAQ. Previous studies validating the MAQ^22,23^ had diverse samples (proportion of women ranging from 42 to 58%, mostly with a high level of education), with no effect on the calculated validity.

Similar to the original authors^22^, we excluded underaged patients from our population, which would probably have affected the validation. Further research is needed to validate the MAQ in this population, as there is no consensus on reducing meat consumption in this age group^54^.

Like in the first validations studies, we did not perform reproducibility and stability over time of the MAQ. These data should be evaluated in future studies.

We did not ask about meat consumption, because it has been correlated with the MAQ score already^22^. However, there is no definitive evidence that it replaces the intake measure. It would also have been beneficial to ascertain how the MAQ correlates with the intakes of different types of meat in our specific population. Further studies are needed to adress this purpose.

In the cognitive interview phase, healthcare professionals were over-represented (7 out of 11). Health professionals may have a higher level of comprehension than other participants, due to better health literacy. However, we did not observe any difference in the level of understanding between participants who were healthcare professionals and those who were not, probably due to the simplicity of the questions of the MAQ.

Finally, as the original authors, we did not make any difference between red and white meat in the questionnaire. This could result in an oversimplification that considers meat as a whole, whereas meat sub-categories are likely to have varying impacts on individual and environmental health.

### Perspectives

From a planetary health perspective, the validation of the French version of the MAQ makes it possible to consider and encourage its use to explore adult patients’ attachment to meat in general practice.

The MAQ discriminates between patient groups effectively^22,23,51,55^. The MAQ is also effective in measuring and predicting people’s motivations and intentions to change meat consumption^22,23,51^. It is therefore an effective research tool, alone or in combination with other scores, for identifying groups with common characteristics with regard to reducing meat consumption (in terms of motivations, barriers and intentionality).

Further studies could be carried out to verify its validity in other French-speaking populations, its reproducibility and its stability over time. Research is also needed to validate its application in underaged patients, whose specificities will probably require the MAQ to be adapted or completed by their parents^56^.

Among the various tools designed to describe attitudes toward food, the Food Neophobia Scale (FNS) is a validated and widely used tool which measures the personal reluctance to accept and/or enjoy new or unfamiliar foods^57,58^. A higher food neophobia (FN) is associated with a lower intake of fruit and vegetables in children and adults^58^. A higher FN represents also a significant barrier to a balanced and healthy diet, such as the Mediterranean diet. On the other hand, a lower FN is associated with a higher acceptance of plant-based meats^58,59^. The combination of FNS and MAQ could prove an effective method of predicting the acceptability of vegetarian alternatives, thus aiding the design of appropriate interventions^59^ and providing valuable insight into meat attachment profiles. For instance, a person with a moderate attachment to meat and a low FN would likely benefit more from an intervention focused on replacing meat with plant-based alternatives than someone with a high FN.

In addition, given that the MAQ questionnaire was developed by studying people’s obstacles and incentives^60^, it aligns closely with the concerns they might have. Its simplicity makes it a useful tool for GPs and health professionals to explore representations of meat-based diets and planetary health. Completing the MAQ in waiting rooms could initiate discussions during consultations. As nutrition becomes a public health concern^3,61^, the MAQ could also be an interesting gateway to a broader nutritional approach.

Finally, from a public health perspective, the MAQ would make it possible to better target the profiles of hedonism, affinity, entitlement and dependence on meat products in the general population. This could be a first step towards developing appropriate population-based strategies to reduce meat proportions in the French diet.

## Conclusion

This study is part of a global health dynamic. Reducing meat consumption is a cobenefit for both individual and environmental health.

We translated and validated the French version of the MAQ in a population of general practice patients. We obtained a 17-item and 4-dimensional questionnaire.

The French version of the MAQ has various possible applications. The use of the French version of the MAQ could fit various strategies aimed at reducing meat consumption. At the GP practice, the MAQ could be used by GPs, medical assistants, public health nurses or advanced practice nurses to explore their patients’ representations to encourage behavioural change. From a public health perspective, the MAQ could be used to better target profiles of hedonism, affinity, entitlement and dependence on meat products in the general population, a preliminary step toward developing appropriate population-based strategies to reduce the proportion of meat products in the diet of the French population.

## Electronic supplementary material

Below is the link to the electronic supplementary material.

Supplementary Material 1

Supplementary Material 2

Supplementary Material 3

Supplementary Material 4

Supplementary Material 5

Supplementary Material 6

## Data Availability

The data that support the findings of this study are available from the corresponding author (HM) upon reasonable request.
