# Supplementary material for: Translation and validation of the meat attachment questionnaire (MAQ) in a French general practice population
Source: Sci Rep. 2025 Jan 18;15:2372. doi: 10.1038/s41598-025-86270-x (PMC11742934; doi:10.1038/s41598-025-86270-x)
Supplement: Supplementary file 1 — Supplementary Material 1 [file 41598_2025_86270_MOESM1_ESM.docx]

Introduction:

"As part of our thesis, we hypothesize that the way we eat can influence health and the environment, and in this context we are working on a questionnaire exploring food, and meat in particular, comprising 16 questions.

We're interested in what you understand about the questionnaire - there are no right or wrong answers.

Before we start, do you know anything about cooking and food? What role does it play in your life? "

Then: "I'm going to ask you a few questions about yourself first, if you agree":

-Age :

-Sex:

-Occupation :

-Main place of residence :

-Education :

-Average meat consumption (per week) :

Red :

White :

Response and coding methods: (please explain)

| **Response rating scale** | **(Lickert)** |  |
| --- | --- | --- |
| A | Strongly disagree |  |
| B | Disagree |  |
| C | Neither disagree or agree |  |
| D | Agree |  |
| E | Strongly agree |  |
| **Coding** |  | **Questions (Probing)** |
| 1 | Appropriate answer and exploration | Why did you answer like that ? |
| 2 | Ambiguous answer | How do you understand the meaning of… ? |
| 3 | Redundant answer | Do you find this question almost similar to another question i have asked you ? |
| 4 | Offensive answer | Do you feel offended by this question ? |
| 5 | Enlightening answer |  |
| 6 | Qualified answer | What do you mean by … ? (depending on the subject) |

Start the questionnaire

**For each question**:

"Can you tell me what you understand about this question and answer it in the way I have explained to you, using your own words, out loud?"

Then targeted questions based on the questions and answers (coded from 1 to 6).

Summing up:

Here's what I've taken from what you've said (summary), do you agree with it/do I accurately reflect your thoughts/opinions?

Do you have anything to add?
