# Supplementary material for: Translation and validation of the meat attachment questionnaire (MAQ) in a French general practice population
Source: Sci Rep. 2025 Jan 18;15:2372. doi: 10.1038/s41598-025-86270-x (PMC11742934; doi:10.1038/s41598-025-86270-x)
Supplement: Supplementary file 2 — Supplementary Material 2 [file 41598_2025_86270_MOESM2_ESM.docx]

**1: Appropriate response**. The response was considered appropriate if the participants answered without hesitation on a Lickert scale.

**2: Ambiguous response.** The answer was considered ambiguous if the participants could not make up their minds, either because they had difficulty understanding the question or because the question had a double meaning.

**3: Redundant answer.** The respondent answers the same thing as a previous answer or considers that they have already answered the question.

**4: Offensive answer.** The respondent feels offended by the question and expresses this verbally.

**5: Interesting answer.** The answer sheds light on the meaning given to the question, either supporting it or providing elements to modify it correctly.

**6 : Qualified answer**. The respondent does not know how to answer the question because it varies according to the circumstances.
