# Supplementary material for: Translation and validation of the meat attachment questionnaire (MAQ) in a French general practice population
Source: Sci Rep. 2025 Jan 18;15:2372. doi: 10.1038/s41598-025-86270-x (PMC11742934; doi:10.1038/s41598-025-86270-x)
Supplement: Supplementary file 3 — Supplementary Material 3 [file 41598_2025_86270_MOESM3_ESM.docx]

1/ Eating meat is one of life's good pleasures.

2/ Nothing can replace meat in my diet.

3/ Because of our place in the food chain, we have the right to eat meat.

4/ I feel bad about eating meat.

5/ I love meals with meat.

6/ Eating meat is disrespectful of life and of the environment.

7/ Eating meat is an indisputable right of every person.

8/ Nothing beats a good steak.

9/ A meat-free diet would suit me just fine.

10/ I love meat.

11/ If I couldn't eat meat, I would be weak.

12/ If I were forced to stop eating meat, I would be sad.

13/ Meat makes me think of illnesses.

14/ Eating meat makes me think of the death and suffering of animals.

15/ Eating meat is a natural practice.

16/ Eating meat is an indisputable practice.

17/ I can't imagine not eating meat regularly.

Scoring rules :

1. The range of responses varies from 1 ("strongly disagree") to 5 ("strongly agree").
2. Items 4, 6, 9, 13, 14 have an inverted score, such as 1=5; 2=4; 3=3; 4=2; 5=1.
3. Averages for each dimension and overall score:
   1. **Hedonism:** AVERAGE (items: 1, 5, 8, 10)
   2. **Affinity:** AVERAGE (items: 4, 6, 9, 14)
   3. **Entitlement**: AVERAGE ((items 3, 7) + ((item 15/2) + (item 16/2))
   4. **Dependence:** AVERAGE (items: 2, 9, 11, 12, 17).
4. Final scores for each dimension and the final score range from 1 to 5. Higher scores are predicted by higher attachment to meat consumption.
