# Supplementary figures and images for: Translation and validation of the meat attachment questionnaire (MAQ) in a French general practice population
Source: Sci Rep. 2025 Jan 18;15:2372. doi: 10.1038/s41598-025-86270-x (PMC11742934; doi:10.1038/s41598-025-86270-x)

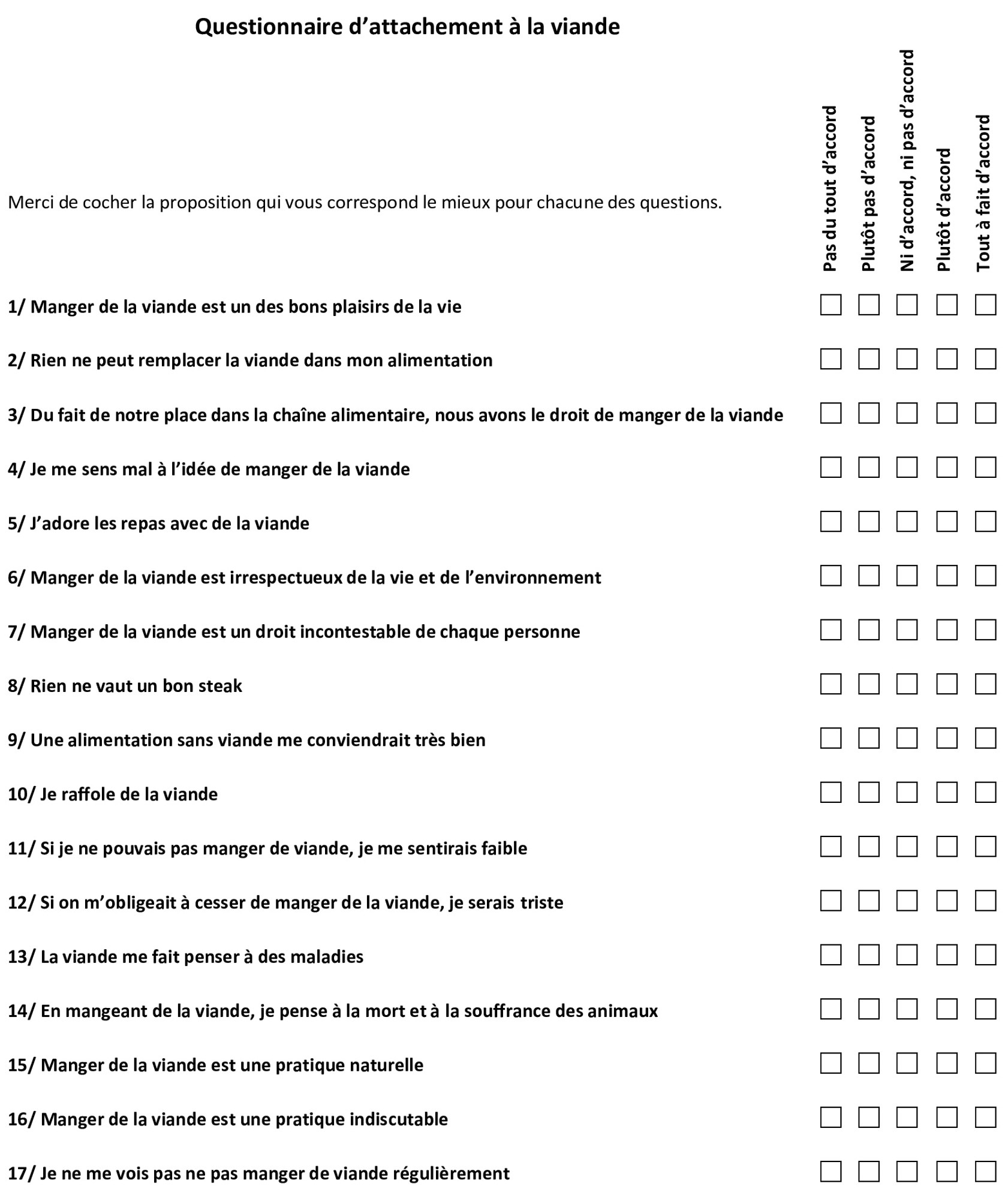

Supplement: Supplementary file 4 — Supplementary Material 4 [file 41598_2025_86270_MOESM4_ESM.jpg]
