# Supplementary material for: Translation and validation of the meat attachment questionnaire (MAQ) in a French general practice population
Source: Sci Rep. 2025 Jan 18;15:2372. doi: 10.1038/s41598-025-86270-x (PMC11742934; doi:10.1038/s41598-025-86270-x)
Supplement: Supplementary file 5 — Supplementary Material 5 [file 41598_2025_86270_MOESM5_ESM.docx]

**1.Leading to the main changes**

Redundant" coding

**-Item 5:** "I love eating meat" (V1) with items 1 and 10:

● B1.1: "this question [...] is almost similar to the question about the pleasure of eating meat" (in relation to item 1).

● B2.1: "[this question is almost similar to the question] “is eating meat one of life's good pleasures" (in relation to item 1).

● A1.1: "It's really a hyper-important pleasure, it reminds me of item 5 but one degree higher" (in relation to item 10)

● C1.1: "This item reminds me of question 5, I put it in the same basket even if the word “raffoler” *(in French)* is a little stronger" (compared to item 10)

***This led us to change item 5 to "I love meals with meat" (V2).***

Ambiguous coding :

**-Item 15:** "eating meat is a natural and indisputable practice" (V1).

● B1.2: "Natural I would answer yes, indisputable I would answer disagree".

● B2.2: "[The words natural and indisputable] are not the same. Indisputable is a bit too much of a word, so I'll take out indisputable."

● C1.2: "Natural practice means that it's something we've always done and yes we've always done it. There's a balance, so yes it's natural, we agree. There will never be a world where no one eats meat, but that's not indisputable. For me, indisputable and incontestable are the same thing, implying that we can't debate it. By that I mean that it's difficult to change behaviour, but we should at least be able to discuss it."

● C2: "I would say it's a natural practice because we've always eaten meat but I wouldn't say it's indisputable."

Illuminating coding:

**-Item 15:** "eating meat is a natural and indisputable practice" (V1)

● A3: "natural practice yes, as we are omnivores, indisputable no, I separated the two notions".

***This led us to split the two notions "natural" and "indisputable" into two separate items (V2****)*

**2.For information**

Differentiated coding :

**Item 3:** "Because of our place in the food chain, we have the right to eat meat".

● B1.3: "The word right is not the word I would have chosen, it's a legal term and we are analyzing what life is like with a human being and animals, and I don't really see what a legal term has to do with it".

**Item 7:** "Eating meat is an unquestionable right of every person".

● B1.4: "The term right is a legal term [...] and it doesn't seem particularly appropriate to me".

Offensive coding:

**Item 6:** Eating meat is disrespectful of life and the environment.

● A1.3: "It's more negative, it's more judgmental than the others. The term disrespectful is very strong: if you eat meat you kill your planet and others around you, which is certainly true but it's very judgmental and negative, it can be offensive, it puts people on the defensive."

● A2: "Disrespectful of the environment, yes maybe, it's a topical issue. But of life, I don't think so".
