# Supplementary material for: Translation and validation of the meat attachment questionnaire (MAQ) in a French general practice population
Source: Sci Rep. 2025 Jan 18;15:2372. doi: 10.1038/s41598-025-86270-x (PMC11742934; doi:10.1038/s41598-025-86270-x)
Supplement: Supplementary file 6 — Supplementary Material 6 [file 41598_2025_86270_MOESM6_ESM.docx]

1/ Manger de la viande est un des bons plaisirs de la vie.

2/ Rien ne peut remplacer la viande dans mon alimentation.

3/ Du fait de notre place dans la chaine alimentaire, nous avons le droit de manger de la

viande.

4/ Je me sens mal à l’idée de manger de la viande.

5/ J’adore manger de la viande.

6/ Manger de la viande est irrespectueux de la vie et de l’environnement.

7/ Manger de la viande est un droit incontestable de chaque personne.

8/ Rien ne vaut un bon steak.

9/ Une alimentation sans viande me conviendrait très bien.

10/ Je raffole de la viande.

11/ Si je ne pouvais pas manger de viande, je me sentirais faible.

12/ Si on m’obligeait à cesser de manger de la viande, je serais triste.

13/ La viande me fait penser à des maladies.

14/ En mangeant de la viande, je pense à la mort et à la souffrance des animaux.

15/ Manger de la viande est une pratique naturelle et indiscutable.

16/ Je ne me vois pas ne pas manger de viande régulièrement.
